# MR-guided ultrasound-stimulated microbubble therapy enhances radiation-induced tumor response

**DOI:** 10.1038/s41598-023-30286-8

**Published:** 2023-03-18

**Authors:** Evan McNabb, Deepa Sharma, Lakshmanan Sannachi, Anoja Giles, Wenyi Yang, Gregory J. Czarnota

**Affiliations:** 1 Physical Sciences, Sunnybrook Research Institute, Toronto, ON Canada; 2 Department of Radiation Oncology, Sunnybrook Health Sciences Centre, Toronto, ON Canada; 3 Department of Radiation Oncology, University of Toronto, Toronto, ON Canada; 4 Department of Medical Biophysics, University of Toronto, Toronto, ON Canada

**Keywords:** Biophysics, Oncology

## Abstract

High intensity focused ultrasound (HIFU) systems have been approved for therapeutic ultrasound delivery to cause tissue ablation or induced hyperthermia. Microbubble agents have also been used in combination with sonication exposures. These require temperature feedback and monitoring to prevent unstable cavitation and prevent excess tissue heating. Previous work has utilized lower power and pressure to oscillate microbubbles and transfer energy to endothelial cells in the absence of thermally induced damage that can radiosensitize tumors. This work investigated whether reduced acoustic power and pressure on a commercial available MR-integrated HIFU system could result in enhanced radiation-induced tumor response after exposure to ultrasound-stimulated microbubbles (USMB) therapy. A commercially available MR-integrated HIFU system was used with a hyperthermia system calibration provided by the manufacturer. The ultrasound transducer was calibrated to reach a peak negative pressure of − 750 kPa. Thirty male New Zealand white rabbits bearing human derived PC3 tumors were grouped to receive no treatment, 14 min of USMB, 8 Gy of radiation in a separate irradiation cabinet, or combined treatments. In vivo temperature changes were collected using MR thermometry at the tumor center and far-field muscle region. Tissues specimens were collected 24 h post radiation therapy. Tumor cell death was measured and compared to untreated controls through hematoxylin and eosin staining and immunohistochemical analysis. The desired peak negative pressure of − 750 kPa used for previous USMB occurred at approximately an input power of 5 W. Temperature changes were limited to under 4 °C in ten of twelve rabbits monitored. The median temperature in the far-field muscle region of the leg was 2.50 °C for groups receiving USMB alone or in combination with radiation. Finally, statistically significant tumor cell death was demonstrated using immunohistochemical analysis in the combined therapy group compared to untreated controls. A commercial MR-guided therapy HIFU system was able to effectively treat PC3 tumors in a rabbit model using USMB therapy in combination with radiation exposures. Future work could find the use of reduced power and pressure levels in a commercial MR-guided therapy system to mechanically stimulate microbubbles and damage endothelial cells without requiring high thermal doses to elicit an antitumor response.

## Introduction

Ultrasound-stimulated microbubbles (USMB) have been used in numerous therapeutic applications such as thermal ablation, hyperthermia, and drug delivery^1–5^. MR-guided ultrasound stimulation with microbubbles has been used as a therapy in uterine fibroids^6,7^ and bone metastases^8^. There, the microbubbles begin to oscillate and enhance the conversion from mechanical to thermal energy^4^. A limiting factor of that method is that temperature effects must be carefully controlled and monitored during therapy, for which several techniques exist^9–11^. Additionally, correction techniques have been proposed to address baseline drifts in the proton resonant frequency (PRF) method for improved accuracy^12^. Thermal effects are not only seen at the acoustic focal point, but also in the far-field regions and along interfaces^13^. This means temperature mapping must be spatially resolvable and span slices perpendicular to the beam axis.

Work has been done using USMB treatments without the direct goal of inducing heat transfer to tissue^14^. Here, the focus is on using ultrasound stimulation with microbubbles to mechanically damage tumor endothelial cells via acoustic energy transfer from the microbubble oscillations^15^. It has been demonstrated in vitro that this action on the endothelial cells sensitizes them to radiation exposure by activation of a ceramide pathway^16,17^. Ceramide production plays a role in the apoptotic response of radiation^18^. The USMB treatment paradigm has been used such that subsequent radiation therapy (XRT) is enhanced, both in vitro and in murine models of prostate, breast, colorectal, and esophageal cancers^19,20^.

One physical difference is the pressure and duration of the ultrasound pulses in USMB treatments differ from those used for therapeutic ablation and hyperthermia. In USMB therapy for radiation enhancement, the pressure and frequency operate mainly at the 500–750 kPa and 500–800 kHz range for 5 mins^21^. This is in contrast to ultrasound ablation where an integrated therapy system may operate at 1.2 MHz and have peak negative pressures exceeding 10 MPa^22^. In hyperthermia, the peak negative pressures may be reduced, but longer durations up to 60 min have been reported^23,24^.

The goal of this work was to build on the results of preclinical therapy systems using a commercial MR-guided therapeutic system containing a multi-element ultrasound transducer. A hyperthermia system configuration was used and a needle hydrophone calibration mapped the input power levels to the desired peak negative pressures used in previous USMB therapies. The integrated setup was used with MR image guidance to plan therapy volumes and to monitor the changes in temperature in prostate cancer tumors using a rabbit model. We hypothesized that temperature changes would not deviate within the tumor or in far-field regions as the peak negative pressure delivered should be lower than that of ultrasound ablation and hyperthermia therapy. USMB therapy was delivered as a single treatment exposure, and in combination with radiation exposures. Finally, treatment response was measured using TUNEL assay analysis to compare the amount of tumor death to previous work using a similar USMB method with a single element ultrasound transducer combined with radiation.

## Methods

### Animal handling and cell preparation

The reporting of this study confirms to ARRIVE 2.0 guidelines. All animal experiments were approved by the Sunnybrook Research Institute Animal Care Committee (SRI ACC, protocol 539). Animal utilization was consistent with guidelines from the Canadian Council on Animal Care (CCAC). Thirty New Zealand male white rabbits (Charles River Laboratories, Montreal, QC, Canada) were injected after reaching a minimum weight of 2 kg with human derived PC3 tumor cells. Tumors grew to 1.5–2.0 cm. Information on animal housing, medications, and cell preparations are described in Supporting Information.

### USMB treatment

**Figure 1:**
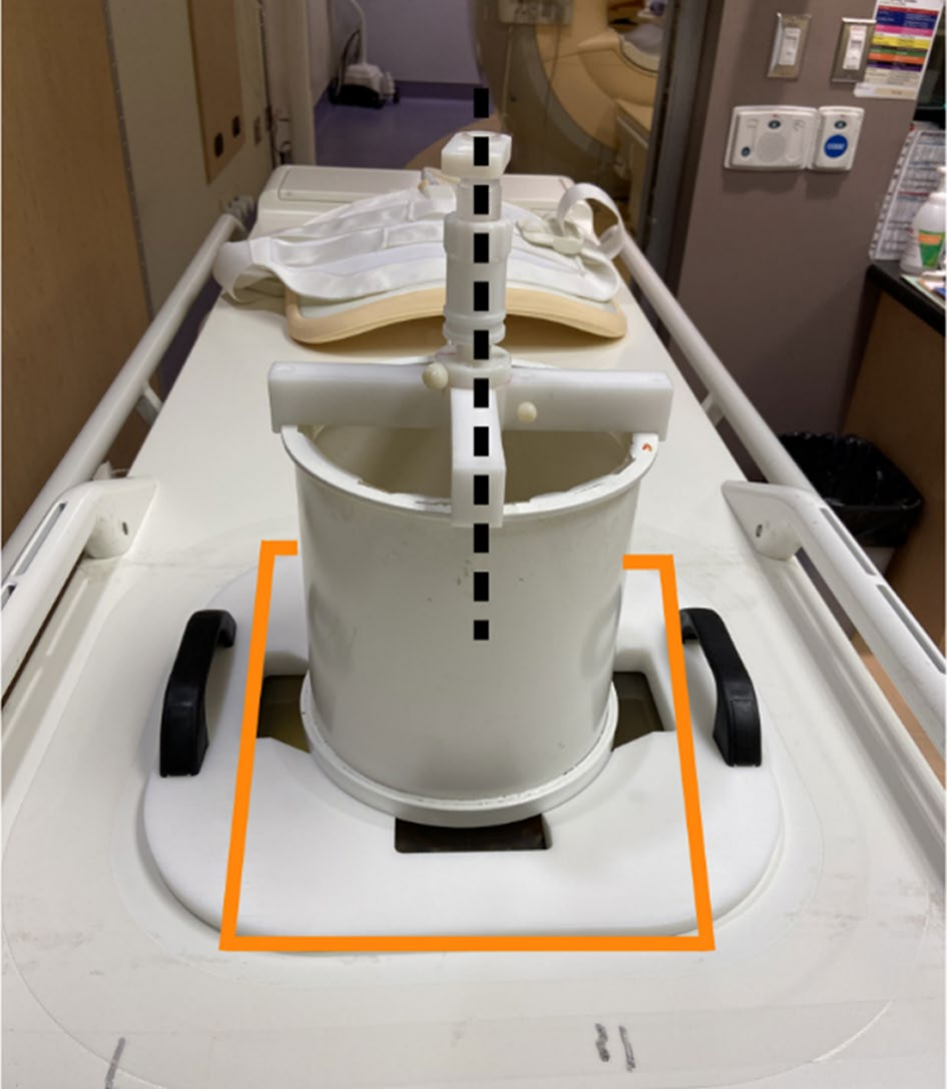
A custom build plastic housing was installed on a hollowed Sonalleve quality control phantom. The dotted black line indicates where a needle hydrophone and wiring could be threaded into degassed water 6 cm above the window membrane (highlighted in yellow).

All experiments were performed using a clinical MR-HIFU system (Sonalleve v2, Profound Medical, Toronto Canada) which was integrated with a 3 T MRI scanner (Achieva, Philips Healthcare, Best, Netherlands). The clinical uses for Sonalleve application involve uterine fibroid ablation, which uses focused ultrasound pulses at 1.2–1.4 MHz and 60–200 W while acquiring rapid MR thermometry images to monitor temperature changes within a treatment area until ablative thermal doses are reached^25^. A series of modifications were used to reduce the mechanical index and induced tissue heating. First, a system-wide calibration mode was obtained by the manufacturer which detuned the operating frequency to 800 kHz and deactivated temperature feedback from the treatment area. The latter was necessary to pulse the ultrasound at fixed acoustic powers and durations. Similar configuration using frequencies under 1 MHz have been previously reported in mild hyperthermia experiments^26^. Secondly, the system was further calibrated to create a peak negative pressure of approximately 750 kPa to be consistent with previous experiments for USMB therapy. A spare Sonalleve quality control phantom, nominally used to verify the heating locations in a gel-like medium, was hollowed of the material and filled with deionized, degassed water^27^. A custom plastic cross was mounted on top of the phantom housing to fit a fiber-optic hydrophone in the centre of acoustic window (Fig. 1). This was initially imaged with the MR system without a hydrophone, and planning images were sent to the Sonalleve workstation. A treatment cell was placed 6 cm above the acoustic window in the center of a coronal plane and window membrane where needle tip of the hydrophone would be placed. The Sonalleve bed was pulled out of the magnet bore, and a needle hydrophone (Precision Acoustics, Dorchester, United Kingdom) was threaded into the plastic housing to verify the pressure as a function of input power to the transducer.

**Figure 2:**
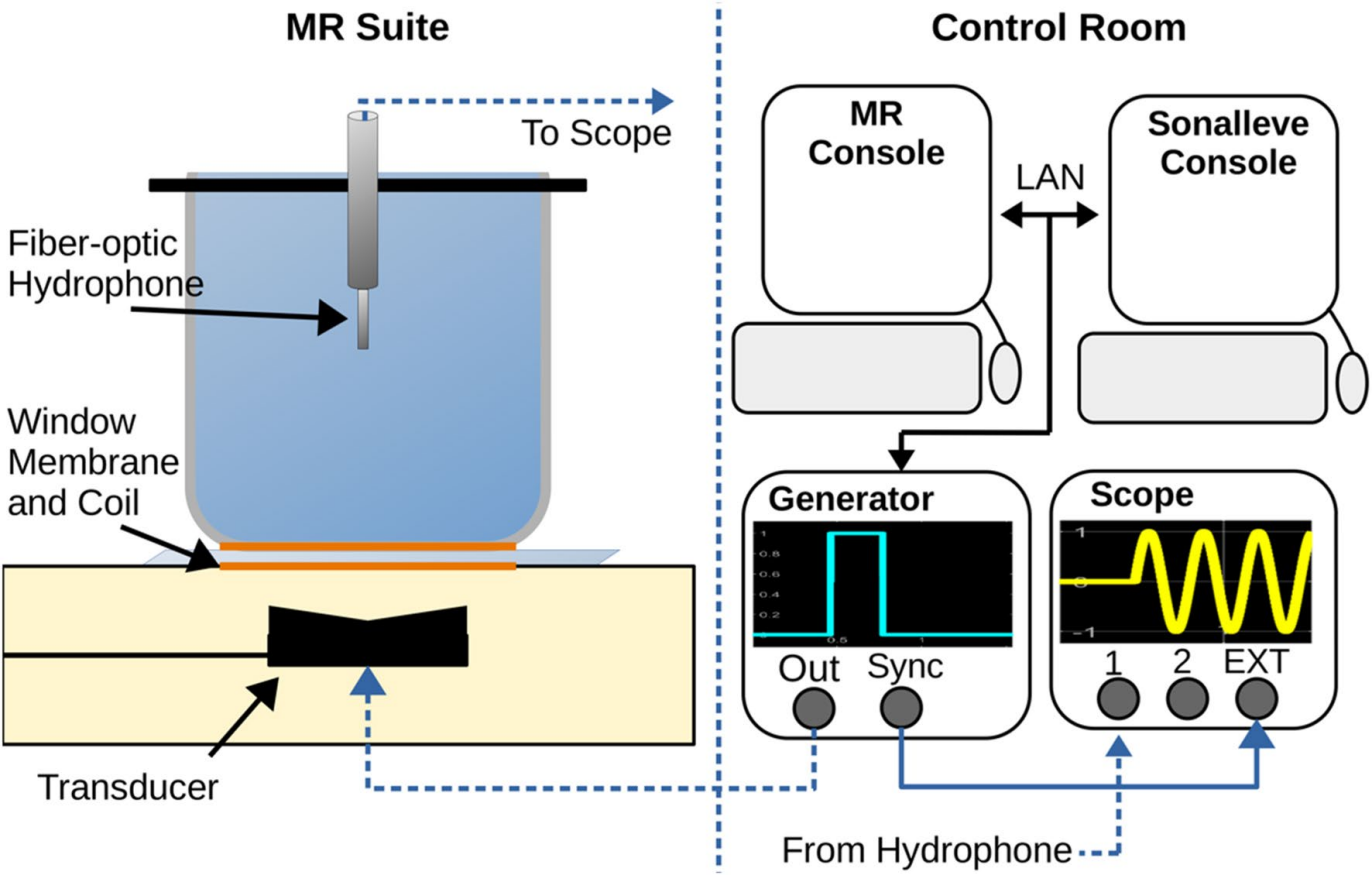
(Left) The MRI suite contains the Sonalleve treatment bed, ultrasound transducer, quality control phantom, and needle hydrophone. The hollowed phantom is filled with deionized, degassed water and acoustically coupled to the window membrane. (Right) The planning images are sent to the Sonalleve console where a treatment cell was placed 6 cm above the membrane. The external trigger function is used to set the trigger approximately 90 µs after the waveform generator burst to avoid measuring electrical coupling of the incident pulse. Dashed lines indicate wiring through separate rooms.

A calibration curve was obtained measuring the peak negative pressure as a function of the acoustic power using an incident wave from Sonalleve and measured with a digital oscilloscope (Fig. 2). The incident wave was a single 16-cycle burst written to a custom XML file. The electronics cabinet contains a synchronous voltage line that was connected to the external trigger of a digital oscilloscope. The hydrophone output wave could be identified at 90 µs, the approximate delay time for the incident acoustic pulse to reach the hydrophone. The focal point was determined by incrementally moving the transducer using the motor positioning tool. The motor position was moved in 1 mm increments in all three planes until a maximum voltage was reached. At this focal point, the acoustic power was reset and adjusted in 0.25 W increments until a voltage was measured, and then in 1 W increments up to 10 W thereafter.

USMB treatment was programmed to always use an 18 mm treatment cell with the transducer at a fixed location. A treatment cell can insonify a volume by electrically steering the focal area over an axial plane and are used to define planning target volumes^25^. Re-positioning the transducer during therapy was not needed as tumor sizes did not exceed 2 cm in diameter and could fit within one treatment cell. The power in Watts was selected by assuming − 1 dB/cm through the tumor with a desired 750 kPa emitted towards the tumor center. The ultrasound wave was set to 5 ms at a 10% duty cycle. A total of 400 repetitions (0.48 Hz repetitions) were performed for a total treatment time of 14 min.

### Experimental protocol

A 2-channel MR coil provided by the manufacturer and inserted below the window membrane was used for all experiments. A custom-built platform was placed on top of the Sonalleve window membrane, with a Mylar base to acoustically couple a water reservoir, allowing wave transmission above the window membrane. Prior to treatments, the animals were anesthetized (3–5% isofluorane) and gently placed on this membrane with their tumor-bearing legs inside the water reservoir. The animal’s heart rate and oxygen saturation levels were continuously monitored and recorded each 15 min as per institutional protocol. Once sedated and positioned, 37 °C degassed water was added to the reservoir.

MR imaging consisted of T1-weighted and T2-weighted imaging for tumor localization which were used as planning images on the Sonalleve. Additional acquisition parameters are provided in Table 1. Gadolinium enhanced (Gadovist, Bayer, 0.2 mmol/kg) images were acquired to assess tumor vasculature followed by 3 mL saline flush. Prior to treatment, T2*-weighted gradient echo images were acquired as a baseline for MR thermometry. During treatment, the same acquisition was used to dynamically acquire slices at the center of the tumor, and tumor/muscle interface superior to the treatment. Dynamic MR images were collected and magnitude and phase data were reconstructed. The phase data were subtracted using the complex phase subtraction method to calculate the phase difference with respect to the baseline time point^28^. Dynamic temperature changes with respect to baseline were calculated using the proton resonant frequency shift (PRF) method using a shielding coefficient of − 0.01 ppm/°C. Voxel-wise time courses of the temperature differences were calculated in MATLAB R2020a (Mathworks, Natwick, USA). USMB treatment and/or XRT treatment followed for non-control groups. Animals receiving USMB treatments were exposed to 1 mL of microbubbles (Definity Lantheus Medical Imaging, MA USA) diluted with 2 mL of saline. Half of the mixture was injected as a bolus at the start of treatment, and the remaining halfway (7 min) into treatment. The animals receiving XRT were transported under anesthesia and placed inside an irradiator cabinet (Faxitron, Hologic Inc, USA) and received a single 8 Gy dose (200 cGy/min). For animals receiving combined therapies, radiation always occurred after USMB treatment within 30 min.

**Table 1:** MRI sequence parameters. All sequences used the 2-channel window coil. Axial images used LR phase encoding to avoid wrap artifacts from the oil tank housing the ultrasound transducer.

	Planning		Post-contrast	MR thermometry
	Cor T1w GRE	Cor T2w FSE	Ax T1w GRE	Ax GRE
TR (ms)	2.7	3000	2.8	140
TE (ms)	1.5	80	1.5	12
BW (Hz/Px)	1750	153	1446	1308
ETL	–	14	–	–
ESP (ms)	–	10.7	–	–
FA	7	90 / 120	7	30
NSA	2	1	2	2
Dynamic phases	–	–	–	11
Resolution (mm)	1.5 × 1.5 × 1.5	1.0 × 1.0 × 1.5	1.2 × 1.2 × 2.0	1.2 × 1.2 × 2.0
FOV (mm)	220 × 238 × 120	300 × 158 × 50	300 × 154 × 40	180 × 180 × 12
Mode	3D	2D interleaved	3D	2D sequential
Scan time (min)	1:48	2:10	0:18	1:25 × 12

### Treatment evaluation

The animals were euthanized under anesthesia with sodium pentobarbital 24 h after therapy, and veterinary technicians excised the tumor that was dissected in half. One half was frozen, and the other fixed was in 10% acetate buffered formalin (Fisher Scientific Canada, Ottawa, Ontario, Canada) for up to 48 h to five days at room temperature. The fixed samples were incubated at 4 °C for 48 h, after which the samples were transferred to 70% ethanol. Further, samples were embedded in paraffin and sectioned to 5 mm by placing it on glass slides for various histology stains. Samples were also stained using hematoxylin and eosin (H&E) staining to visualise cellular morphology, and terminal deoxynucleotidyl transferase dUTP nick end labeling (TUNEL) staining was used to detect apoptotic regions. The samples were stained at the Pathology Research Program, University Health Network, Toronto, ON, Canada.

Whole mount stains were imaged under magnification and digitized (TissueScope, Huron Digital Pathology, St. Jacobs, ON, Canada). Tumor regions of interest (ROIs) were drawn on the H&E images and registered to TUNEL stains to remove any skin and muscle tissue from analysis. Tissue slides were unblinded. Colour deconvolution was performed on TUNEL images in ImageJ to isolate DAB + channels^29,30^. These channels were thresholded and the percent positive staining was calculated as the number of pixels remaining after thresholding divided by the number of pixels in the tumor ROI. Finally, once all ROIs were drawn, colour deconvolution was automated in Python (https://python.org) and all quantitative values were calculated using the same parameters.

### Statistical analysis

Regression analysis and one-way analysis of the variance (ANOVA) were calculated using a significance level α = 0.05 for all tests. Linear regression first fitted the voxel-wise time courses in MR thermometry data with respect to time from baseline. A second linear regression fitted the same voxel-wise time course to only the mean of the data. A non zero trend was defined as any regression with a statistically significant F-statistic when comparing the two nested models. ANOVA used the mean of each treatment group. Multiple comparisons (Dunnett’s method) tested the combined treatment group against all other groups.

## Results

### Ultrasound pressure

**Figure 3:**
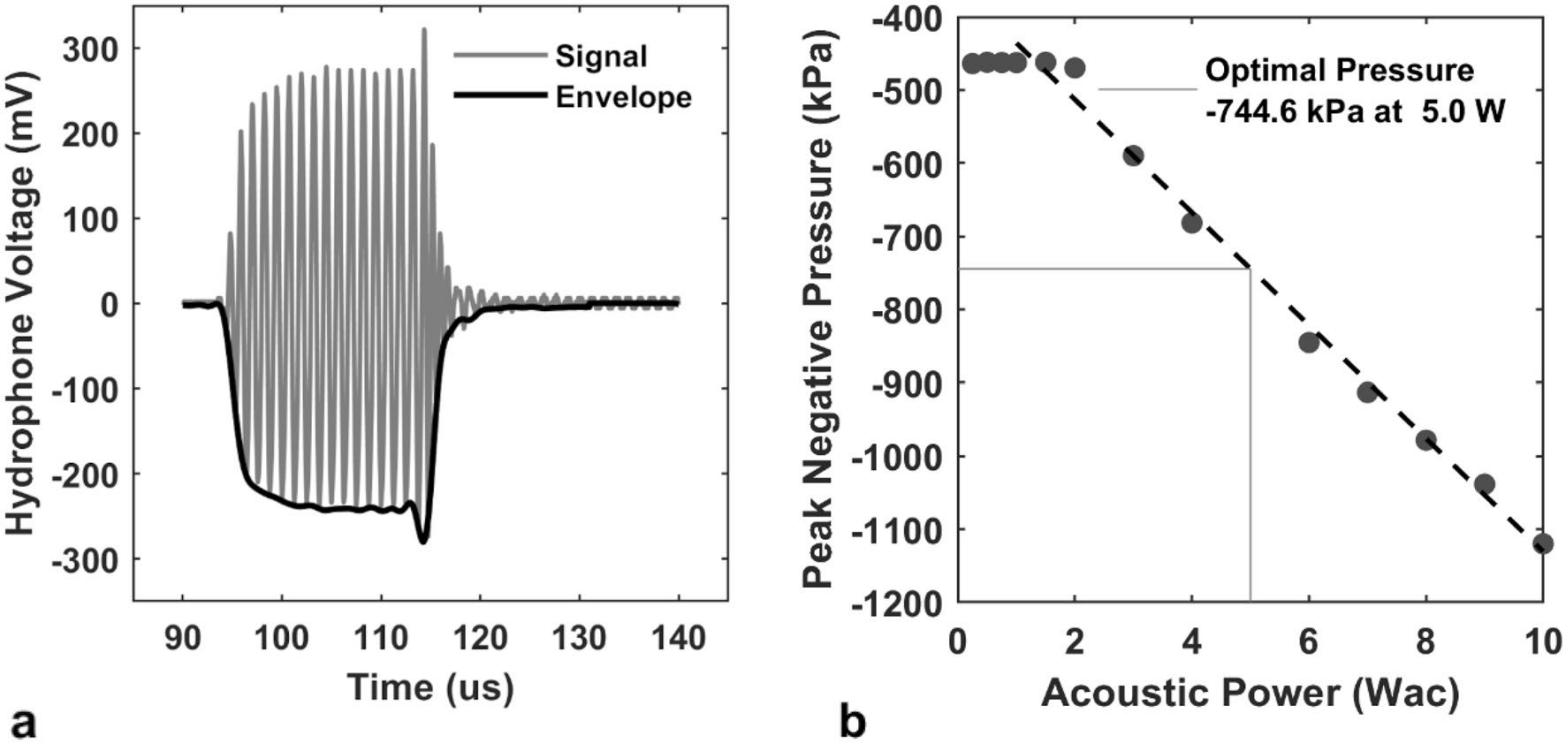
Detuned 800 kHz Sonalleve hydrophone measurements at the focus. (**a**) Manual 16-cycle bursts were sent to the Sonalleve transducer, and the subsequent voltage was measured approximately 90 µs after using a manual trigger. The peak negative pressure was calculated as the plateau voltage after filtering through an envelope detector multiplied by the hydrophone sensitivity factor. (**b**) Peak negative pressures plotted as a function of acoustic power on the Sonalleve console. A negative linear trend was observed between 1.75 and 10 Wac (n = 9 points). Without any attenuation, approximately 750 kPa peak negative pressure can be achieved using 5 Wac.

The incident 16-cycle acoustic pulse measured with the digital oscilloscope was filtered with an envelope detector and converted into peak negative pressures (Fig. 3). The displayed signal and envelope were representative of the received hydrophone voltages and signal characteristics, including a rise time of 3–4 cycles to reach a steady state amplitude followed by a positive voltage spike at the end of the acoustic pulse before oscillating towards 0 mV. In all cases, the negative side of the envelope was used. No voltage was observed until the input acoustic power reached 1.75 W, however voltage response was strongly linear from 1.75 to 10 W (n = 9, r = 0.996). A linear regression model was fitted to predict the acoustic power needed for − 750 kPa at the focus. The fitted slope and intercept were − 359 kPa/W (95% CI: − 390 to − 327 kPa/W) and − 77 kPa (95% CI: − 82.4 to − 71.9 kPa) respectively. As the Sonalleve can only deliver acoustic power rounded to the nearest in 0.5 W, the closest acoustic power is achieved at 5 W, and was less than 1% deviation from the desired pressure.

### Treatment monitoring

**Figure 4:**
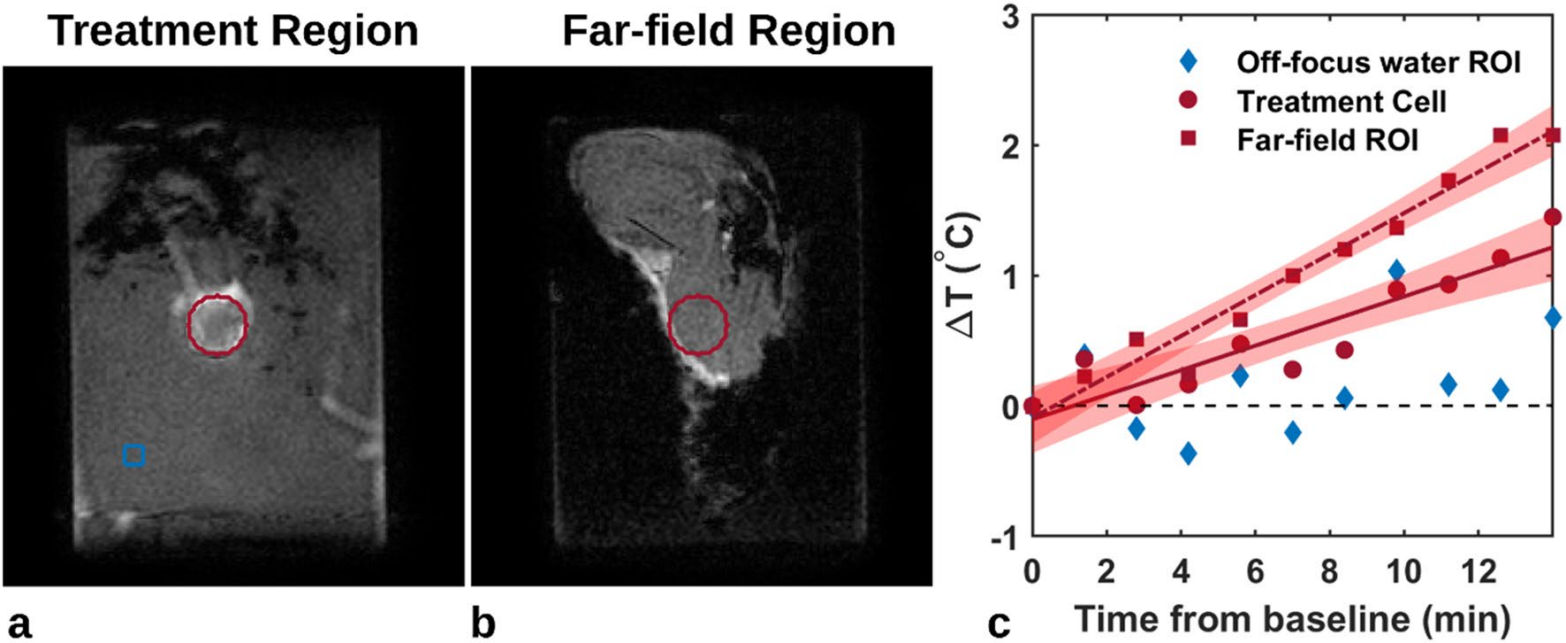
(**a**) Coronal plane perpendicular to the beam axis with an 18 mm treatment region (red) used to define the therapy cell on the Sonalleve and as a mask for voxel-wise temperature mapping; an off-focus region in the water bath for comparison (blue). (**b**) Coronal plane translated anteriorly 1.5 cm to define the far-field region at the tumor and muscle interface. The same treatment area is shifted to the far-field region. (**c**) Mean temperature mapping signals (n = 377 voxels in ROI) within the therapy cell (red circles) and far-field region (squares). Regression was statistically significant in both regions. Shaded areas represent 95% confidence intervals of the slope. The off-focus water region (blue diamond) was not significantly different than zero.

Mild temperature increases were observed in both in the treatment region and in the far-field region over the treatment duration (Fig. 4). The 1.8 mm treatment region is shown in the center slice of the tumor, as well as the same region superimposed on the slice containing muscle distal to the tumor bed. In the example shown, the proportion of voxels reaching a statistically significant trend were 73% (95% CI: 68–77%) and 75% (95% CI: 71–80%) in the treatment focus and far-field regions, respectively. The voxel-wise slopes (n = 377 for each ROI) in both regions were significantly different from zero, indicating an increasing temperature during therapy with maximum temperatures of 1.50 °C and 2.39 °C after 14 min of therapy. Not only does the temperature increase most in the far-field region, but the shaded confidence limits are also narrower around the fitted slope. This coupled with the higher proportion of voxels reaching a statistically significant trend suggest a greater uniformity and likelihood of temperature increases in the 18 mm region above the tumor. A reference region in the water reservoir was added to measure any linear drift, which did not reach significance (blue diamonds about the zero axis).

**Figure 5:**
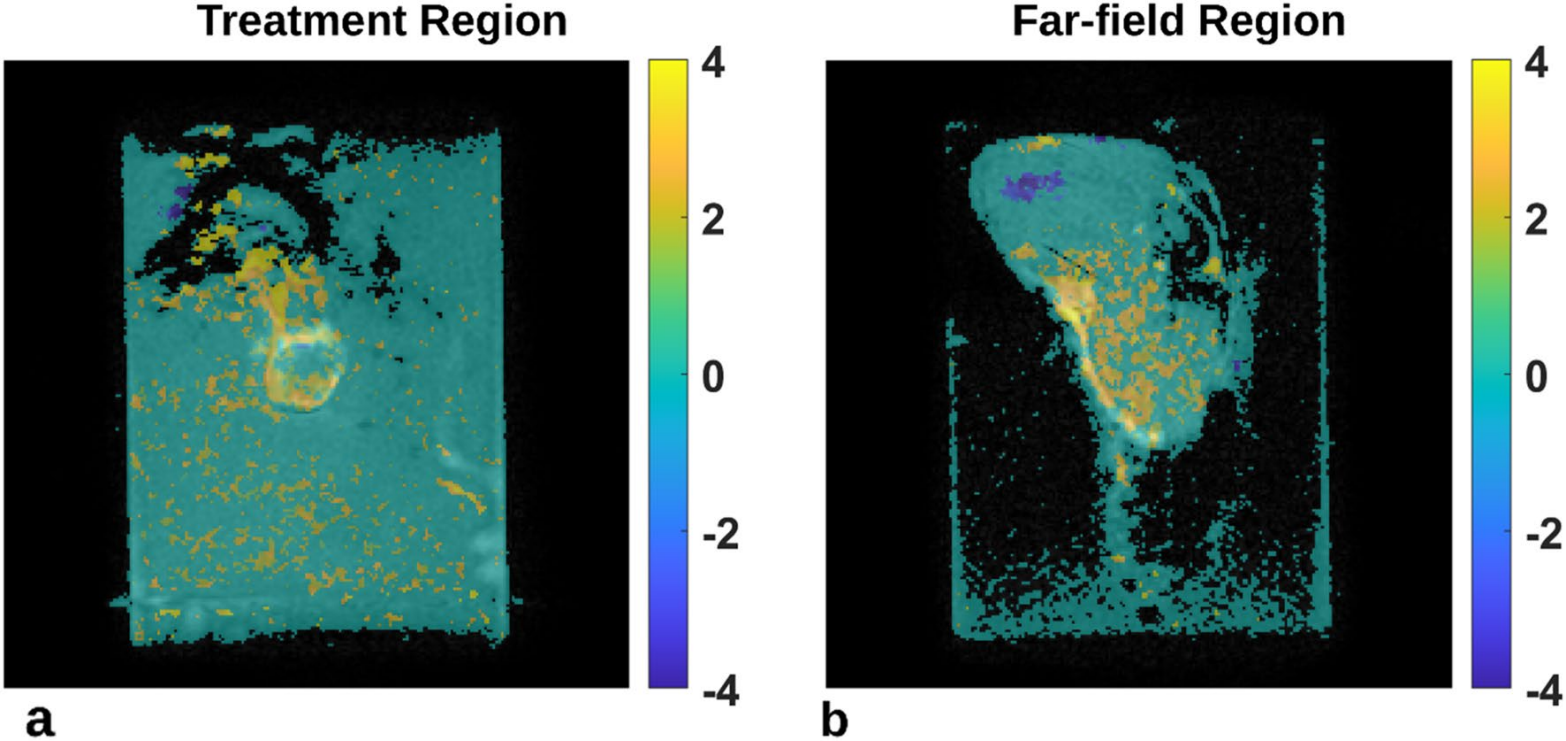
(**a**) Maximum temperature map overlaid on coronal plane perpendicular to the beam axis mid tumor and (**b**) in the far-field region. Values represent the temperature model taken at t = 14 min, or the end of therapy. Voxel time courses that did not have a statistically significant linear fit were set to transparent voxels. Parametric maps were median filtered (3 × 3 kernel) for visual display.

Spatial uniformity of temperature effects differs between the treatment focus and far-field regions (Fig. 5). Voxel-wise time courses were first fit to a linear model and if the fit was statistically significant, the model was evaluated at time t = 14 min to estimate the maximum temperature resulting from therapy, otherwise the voxel was set to a transparent value. In the focus region, there exists spatial grouping of pixels suggesting certain intratumor areas may have better capacity in dealing with induced heating. However, the maximum temperature reached from each voxel-wise time courses is more uniform (Fig. 5b).

The maximum temperature increase was calculated from a linear model and averaged spatially over the 18 mm treatment area of both regions in eleven animals receiving USMB therapy (Table 2). A statistically significant increase was seen in both regions with maximum temperature increases of 1.22 °C (*p* = 0.006) at the treatment focus and 1.61 °C (*p* = 0.04) at the far-field region, respectively.

**Table 2:** Summary statistics of the maximum temperature after 14 min of therapy in the treatment and far-field regions across the treated animals.

	Treatment cell (°C)	Far-field (°C)
Mean	1.22*	1.61*
SD	1.16	2.32
Median	1.50	2.50
	*p* = 0.006	*p* = 0.044

Two-sided, two sample *t*-test; *df* = 10.

### Treatment evaluation

**Figure 6:**
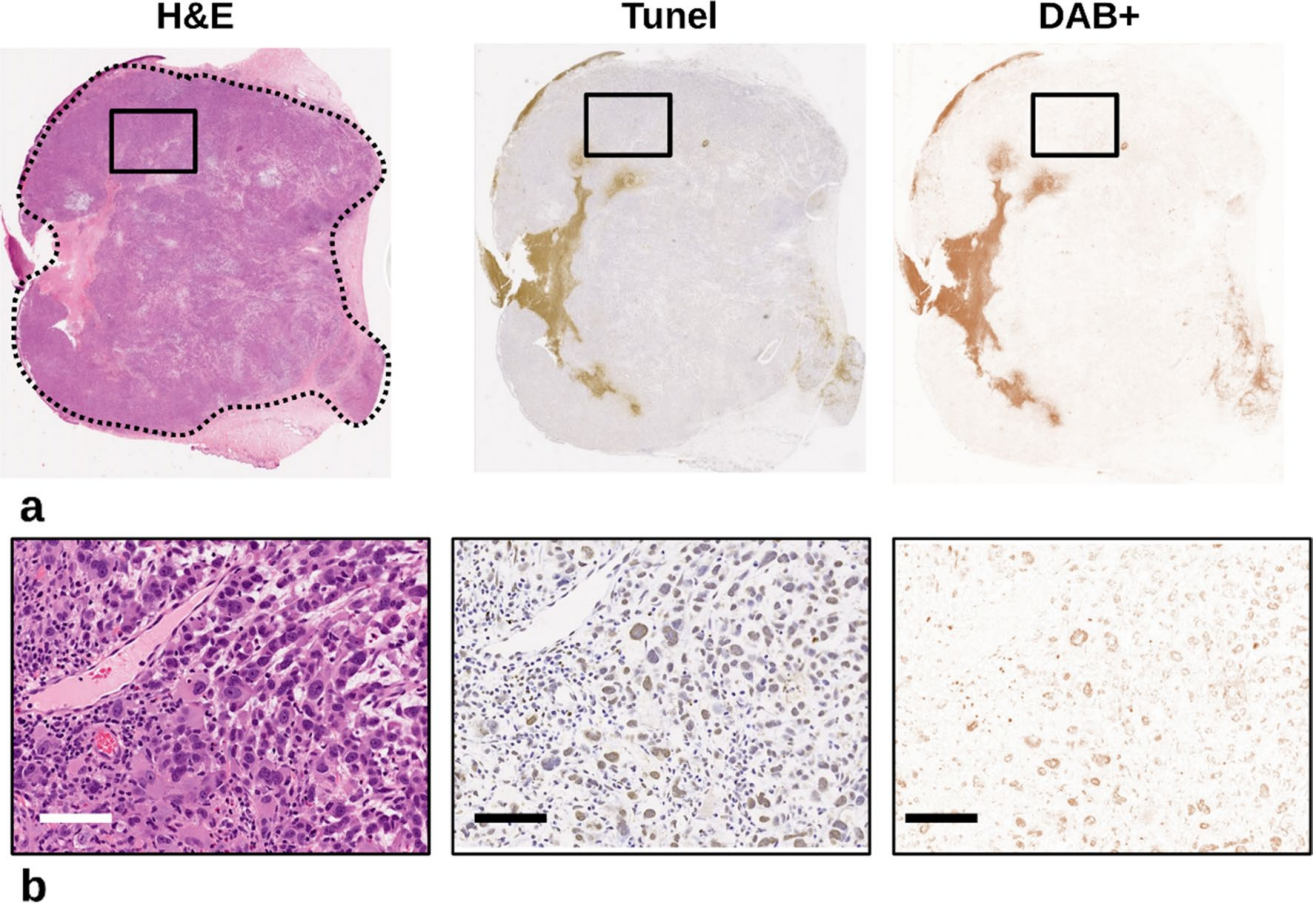
(**a**) Light microscopy of whole mount slides displaying H&E, TUNEL, and the DAB + channel respectively. Regions of interest were drawn (dashed curve) on H&E slides avoiding skin and muscle tissue. Color deconvolution separated the TUNEL stains into channels where the DAB + channel was isolated, and the percent positive staining can be calculated as the ratio of included pixels divided by the number of pixels in the ROI. (**b**) Magnified views (25X; 0.4 × 0.4 µm) of the highlighted regions (black box). Color deconvolution shows good agreement with the brown positive stain in TUNEL stain and absence of background stain. Scale bar represents 160 µm.

Tumor regions of interest were traced on whole mount light microscopy H&E slides and transferred to TUNEL slides for color deconvolution (Fig. 6). Whereas, the H&E slides were used to identify cell characteristics such as binucleation and nuclear fragmentation, the TUNEL slides stained cells in the apoptosis phase. These were separated out and quantified by color. In the 25× magnified views (Fig. 6b), even cell clusters with mixes of both stains were separated so that only the brown positive stained regions can be quantified.

**Figure 7:**
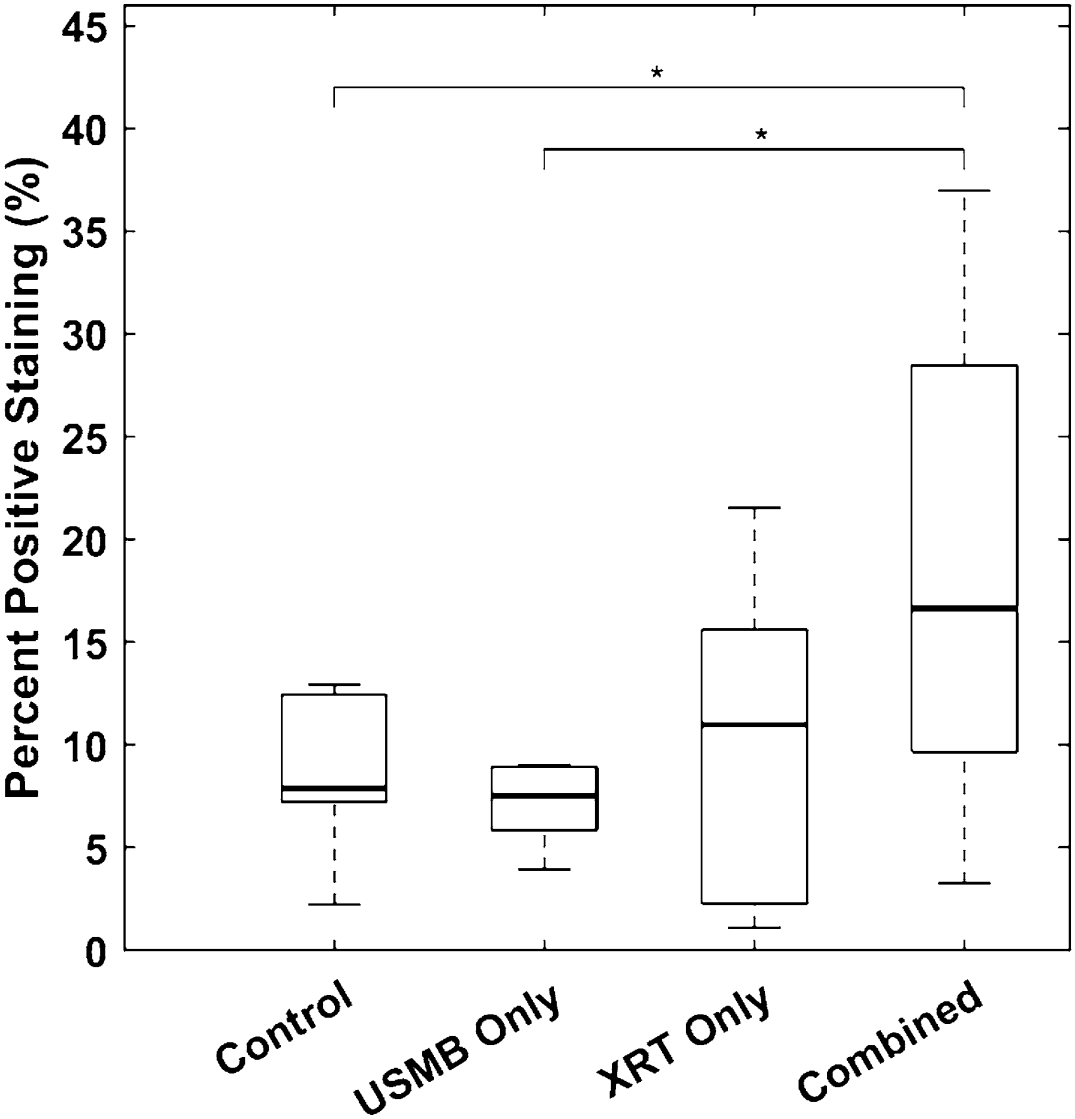
The percent positive staining for untreated controls, USMB-only, XRT-only, and combined treatment groups respectively. Boxplots show groupwise median (bold line), interquartile range (box), and standard deviation (dashed lines). A one-way analysis of variance demonstrated a statistically significant difference between group means (n = 30, *df* = 26, *p* = 0.026). Group-wise multiple comparisons are shown in horizontal lines. The mean differences in percent positive staining between combined and untreated controls, USMB-alone, and XRT-alone groups were 9.68% (*p* = 0.037, 95% CI: 0.47–18.9%), 11.3% (*p* = 0.036, 95% CI: 0.62–22.0%), and 3.99% (*p* = 0.140, 95% CI: − 1.99–18.1%), respectively.

The percentage of positive staining within a single tumor ROIs on TUNEL images revealed differences between the treatment groups (Fig. 7). A one-way analysis of variance between treatment groups demonstrated a statistically significant mean difference (n = 30, *df* = 26, *p* = 0.026). The mean differences in percent positive staining between combined and untreated controls were 9.68% (*p* = 0.037, 95% CI: 0.47–18.9%). The mean differences between combined and USMB-only were 11.3% (*p* = 0.036, 95% CI: 0.62–22.0%). Finally, the mean differences between combined and XRT-only groups were 3.99% (*p* = 0.140, 95% CI: − 1.99 to 18.1%).

## Discussion

The work here demonstrated a clinical MR-integrated HIFU system was able to induce tumor cell death using USMB therapy for the purpose of radiation enhancement. The system used a mild hyperthermia therapy mode, and a calibration was performed such that the peak negative pressures in vivo were consistent with previous in vitro and murine models using single element transducers. Temperature monitoring in vivo demonstrated that temperature changes were overall well below (no heating) the limits used in mild hyperthermia effects. Treatment effect through immunohistochemical analysis was measured 24-h after initial USMB and/or radiation and revealed a significant difference in tumor cell death in the group combining USMB + XRT exposures.

Temperature changes induced in tissue were measured with the PRF MR Thermometry method. Maximum temperature changes treatment occurred in the far-field region, approximately 1 cm distal to the center of tumor. The median group-wise temperature rise was 2.50 °C for animals receiving any method of USMB exposures. This was less than what would be expected for a mild hyperthermia experiment, which aims to hold tissue temperatures above 41 °C^31^. Additionally, in 10 out of the twelve animals, temperatures differences were below 4 °C in all dynamic phases. The two that exceeded this threshold reached average increases of 4.0 and 4.2 °C in the far-field regions, respectively. The longest treatment duration was 14 min compared to other mild hyperthermia studies which may expose tumors to treatment durations ranging from 15 to 60 mins^23,24^. The method here can be beneficial as patient movement is a limitation in image guided focused ultrasound techniques. However, it is still longer than ablative-based techniques whose treatments, though require stricter control in temperature monitoring to avoid burns to healthy tissue in adjacent areas surrounding the tumor, as well as skin in the near-field region.

Combined USMB + XRT results in this work demonstrated a significantly higher tumor cell kill compared to untreated controls or USMB alone^19^. Previous studies using a similar 8 Gy radiation exposure and 3% (v/v) microbubble treatments in a rabbit model differ from the results here. In previous experiments, the USMB therapy was with a single element transducer that generated a planar wave, as opposed to a multi-element transducer using electrical focusing at the tumor. Tumor cell death stained with an ISEL assay demonstrated a higher amount of cell kill, with 20% ± 7% compared to control and 29% ± 8% compared to USMB alone. The lower values found here may result from differences in the percentage of positive staining, and less mechanical stimulation on individual focus points within the tumor. Additionally, as opposed to a planar wave, the focal points here are steered within a treatment cell and are updated based on a trajectory every 50 ms. The amount of mechanical energy transferred to microbubbles at each focus point is less than can be achieved in a plane wave updated at the same rate.

A limitation of this study is the hydrophone used did not have a voltage-pressure sensitivity listed for 800 kHz. The lowest value listed per the manufacturer’s specification was 1 MHz, which had a sensitivity of 216 mV/MPa, which was also used for this study. The sensitivity values ranged from 200 to 250 mV/MPa for all the operational frequencies below 28 MHz. Repeating the power to peak negative pressure calibration calculations with the lowest possible sensitivity of 200 mV/mPa would not change the optimal desired input power used in this study (5 W). Conversely, using 250 mV/MPa sensitivity, the closest input power was 6.5 W in order to reach the desired pressure of − 750 kPa. In this case, the selected input power of 5 W would underestimate the pressure given the clear correlation seen in the data. This would result in a lower potential for induced tissue heating, but at the cost of less treatment effect^21^.

MR imaging was limited by several factors. The oil tank that houses the transducer is below the animal and requires larger field of views, which either limits resolution or requires additional data sampling (e.g., image matrix) which increases image duration. We chose to avoid longer imaging times, as prolonged imaging times risk subject movements and would require subject repositions and additional images to place a new therapy cell. Transitioning to human experiments would likely experience similar resolution versus duration trade-offs since patient movement prior to treatment would cause the therapy cells to be misregistered. Secondly, the signal-to-noise ratio (SNR) is limited by the two-channel receive array below the water reservoir. Since the Sonalleve transducer has less available mobility in anterior–posterior position, the therapy cell remained approximately 6 cm above the window membrane. This means the middle of the tumor was 6 cm away from the acoustic window (and therefore the coil array) causing a SNR loss. This is especially problematic for most quantitative imaging techniques such T2* temperature mapping techniques. Finally, we elected not to use echo-shifting techniques such as principles of echo-shifting with a train of observations (PRESTO) or rapid echo planar imaging readouts which can be more sensitive to B0-variations. These techniques also use higher imaging sampling rates and may exacerbate the loss in SNR^32^.

During the initial calibration procedure, we used a high power, high duty cycle alternative sequence in a dedicated Sonalleve quality control phantom. This was performed at 70 W over 5 min at multiple cell locations. Temperature increases were evident and shaped according to the expected size of the therapy cell. This higher-powered calibration setting, compared to the hyperthermia settings used for USMB exposures, demonstrated that the equipment was correctly functioning with high input power.

The spatial uniformity of temperature changes were non-uniform in tumors as measured by MR thermometry data. The displayed quantitative maps of temperature changes with respect to baseline demonstrated temperature changes can be absent, or up to 4 °C in different areas of the tumor. One concern regarding the spatial distribution of temperature changes is that it was difficult to correlate the average temperature change in the treatment volume or far-field volumes to the amount of tumor cell death. It is clear that certain areas of the tumor had different temperature changes, but what remains unclear is whether these same areas correlated to the TUNEL assay. This study did not perform a registration of slides to imaging location.

In work performed by Lam et al., which held tumors to 40–42 °C, vessel perfusion was assessed through dynamic contrast enhanced (DCE) MRI and intravoxel incoherent motion (IVIM)^33^. The perfusion values were heterogeneous between animals prior to therapy which increased in follow-up imaging. The spatial non-uniformity in temperature seen here may be a result of intratumor vessel distribution and their ability to regulate against induced heating at low ultrasound power. Future work may benefit from using a perfusion-based technique to preferentially target vessels.

## Conclusions

A commercial MR-guided therapy HIFU system was able to treat rabbits bearing PC3 tumors on their hind legs using ultrasound-stimulated microbubble therapy in combination with radiation exposures. The work presented here used a mild hyperthermia system setting, reduced input power of 5 W was able to deliver approximately 750 kPa of peak negative pressure to tumors. Treatments consisted of microbubbles and sonication exposures for 14 min followed by 8 Gy of radiation to the entire tumor. Tumor cell kill was significantly higher in combined USMB and XRT treatment groups. Future work could find use in reduced power levels and ultrasound pressure without the need for dedicated temperature feedback.

## Supplementary Information

Supplementary Information.

## Data Availability

The datasets used and/or analysed during the current study are available from the corresponding author on reasonable request.
