# Supplementary material for: MR-guided ultrasound-stimulated microbubble therapy enhances radiation-induced tumor response
Source: Sci Rep. 2023 Mar 18;13:4487. doi: 10.1038/s41598-023-30286-8 (PMC10024768; doi:10.1038/s41598-023-30286-8)
Supplement: Supplementary file 1 — Supplementary Information. [file 41598_2023_30286_MOESM1_ESM.docx]

**Supporting Information**

*Animal Handling and Cell Preparation*

This study used the following humane endpoints: tumors exceeding 2 cm in diameter, greater than 20% weight loss, lack of feeding, lack of ambulation, and self-mutilation, bleeding, or ulcerations of the tumor area. All animals were euthanized under anaesthesia with intravenous sodium pentobarbitol (Euthanyl) immediately at endpoints.

Animals were evaluated in-house daily by trained veterinary staff and supportive care was given when necessary for minor wounds (analgesia and polytopic antibiotics). Animals were housed individually, provided fresh food and water daily, and all cages are supplied with enrichment items. Daily checks to animal weight, sickness, wounds, excrement, etc. were scored and recorded. Each New Zealand White rabbits received three treatments of enrofloxacin (Baytril) at 5 mg/kg upon arrival and were housed locally in HEPA filtered, positive pressure clean rooms until they reached the appropriate weight range listed (approximately 7 to 10 days). The rabbits were immunosuppressed by daily intramuscular injections of cyclosporine at 50 mg/mL (Sandimmune, Novartis, Dorval, QC, Canada). Prostate cancer (PC3) cells were purchased directly from a vendor (ATCC CRL1435, Manassas, VA, USA).

Cell cultures were incubated at 37 °C and suspended in phosphate buffered saline until. At passages 3, approximately 700 µL of cells were prepared for each animal. Tumor injections were performed under anaesthesia (5% isoflurane) in the right hind leg of the rabbit. Immunosuppression continued daily until tumors grew to 2 cm in diameter or the animal reached an endpoint.
